# Dr. Earl Philip Warren

**Published:** 1912-10

**Authors:** 


Dr. Earl Philip Warren, P. & S., 1902, of Niagara Falls, died of ptomain poisoning following typhoid, Aug. 12, aged 39.

